# Urinary tract infection in cancer patients and antimicrobial susceptibility of isolates in Tikur Anbessa Specialized Hospital, Addis Ababa, Ethiopia

**DOI:** 10.1371/journal.pone.0243474

**Published:** 2020-12-10

**Authors:** Wondewosen Tseagye Sime, Habtamu Biazin, Tamrat Abebe Zeleke, Zelalem Desalegn

**Affiliations:** 1 Department of Microbiology Immunology and Parasitological, St. Paul's Hospital Millennium Medical College, Addis Ababa, Ethiopia; 2 Department of Microbiology Immunology and Parasitological, School of Medicine, Addis Ababa University, Addis Ababa, Ethiopia; Nitte University, INDIA

## Abstract

**Background:**

Urinary tract infections are the most common causes of morbidity and mortality in patients with cancer. The emergence of multiple-drug-resistant (MDR) strains of gram-negative bacteria causing urinary tract infection has become a serious concern in cancer patients. Therefore, the present study aimed to determine the spectrum and antibiotic resistance pattern of bacterial isolates related to urinary tract infections among cancer patients at Tikur Anbessa Specialized Hospital, Addis Ababa, Ethiopia.

**Methods and materials:**

Hospital based prospective cross-sectional study was conducted for three months from January to March 2018 in tertiary care hospital located in the capital city of the country. Gram-negative bacteria isolated from urine specimens from hospitalized patients with cancer were characterized using standard microbiological methods. Modified Kirby–Bauer disk diffusion technique was applied for antimicrobial susceptibility testing in accordance with CLSI 2019 criteria.

**Results:**

Of totally 292 urine samples tested, eighteen (6.3%) were culture positive cases, *Escherichia coli* (44.4%) was the highest proportion isolated uropathogen followed by *Klebsiella pneumoniae* (22.2%) and *Citrobacter diversus* (16.7%). The antibiotic susceptibility results showed meropenem and nitrofurantoin as the most effective antibiotics for *E*. *coli*, *K*. *pneumoniae*, and *Citrobacter diversus* isolates. The rate of multidrug resistant (MDR) isolates were 33.3% (6/18), and meropenem and nitrofurantoin were the most effective antibiotic against MDR isolates.

**Conclusion:**

The study findings showed a significant distribution of MDR gram-negative bacteria which may increase the burden of urinary tract associated infections in cancer patients. Carbapenem (meropenem) can be considered as effective agents to treat MDR cases in our region.

## Introduction

Cancer is a significant cause of death worldwide, and more than half of them occur in developing countries. The most common causes of cancer death are lung, liver, colorectal, stomach, breast cancer, cervical cancer, and leukemia [1]. The new advances in treatment options increased survival rates of cancer patients in the past decades [2]. However, severe immunosuppression as an adverse consequence of these treatment strategies increases the risk of opportunistic infections. Infections are one of the most serious complications and the leading cause of morbidity and mortality in patients with cancer [3]. There are several risk factors for acquisition of infections such as neutropenia, stem cell transplantation, long-term catheterization, and the extensive use of medical devices such as stents, shunts and central venous catheters [3, 4]. One of the most common infection in cancer patients is urinary tract infections (UTIs) [3, 4]. A wide range of bacteria has been reported as a cause of urinary tract infections that among them, Enterobacteriaceae are the most prevalent [4]. The emergence of multiple-drug-resistant (MDR) strains of Gram-negative bacteria causing UTIs has become a serious concern, especially in cancer patients [5]. The incidence of infections caused by multi-drug resistant (MDR) bacteria has been increasing throughout the world [6, 7]. The number and MDR spectrum of microbial infections might increase by the administration of new, stronger immunosuppressive regimens [8].

In recent years, the majority of conducted studies in cancer patients have only focused on blood stream infections, and there is scarcity of information with regards to the overall prevalence of gram-negative bacteria related among cancer patients suspected with urinary tract infection in Ethiopia. Therefore, the present study aimed to determine the spectrum and antibiotic resistance pattern of gram-negative bacteria related urinary tract infections among cancer patients. This information can help clinicians to choose effective therapies and provide good epidemiological profiles to compare our situation with others.

## Materials and methods

### Study area, design, and period

The study was conducted at Tikur Anbessa specialized hospital from January up to March 2018. A prospective hospital based cross-sectional study design was employed.

### Study population

All adult female cancer patients during the study period were considered as a study population.

### Clinical and laboratory data

#### Clinical data

A structured questionnaire was used to collect clinical data and physicians collected the relevant clinical data.

#### Urine samples collection and processing

Early morning 5 ml mid-stream clean catch urine [MSU] specimens was collected using lick proof re-usable sterile plastic containers on the same day of enrolment. All the specimens were processed within an hour of collection.

#### Bacterial isolation

Using calibrated wire loop [0.001 ml] samples were inoculated in to Cysteine Lactose Electrolyte Deficient medium [CLED] agar. After overnight incubation at 37°C for 24–48hours colonies were counted to check significant growth.

Colony counts yielding bacterial growth of 10^5^/ml [A diagnosis of UTI was made when there are at least 10^5^ colony forming unit (CFU)/ml] of urine was regarded as significant for bacteriuria. Colonies from CLED were sub cultured into MacConkey agar and blood agar plates [BAP] [Oxoid, LTD] and incubated at 37°C for 24–48 hours. Identification of bacteria were done using colony characteristics, gram reaction of the organisms and biochemical tests following standard bacteriological procedure [9, 10]. All laboratory procedures were conducted in Black Lion specialized hospital laboratory and the same senior laboratory technologist performed the tests in all the time to avoid professional biases. For contaminated specimens, culture was repeated.

#### Antimicrobial susceptibility testing (AST)

All identified pure bacterial isolates were subjected to in vitro susceptibility testing by Modified Kirby Bauer as previously described [11–13] for 12 antibiotics such as amoxicillin-clavulanic acid (Augmentin) (20μg), ampicillin (30μg), ciprofloxacin (5μg), Nitrofurantoin (300ug), Ceftazidime (10μg), gentamicin (10 μg), co-trimoxazole (Trimethoprim-Sulfamethoxazole) (25 μg), Meropenem (10μg), Cefepime (30μg), Piperacillin-Tazobactam (16μg), Chloramphenicol (30μg), and Ceftriaxone (10μg). A suspension of a pure colony from each confirmed culture isolate was prepared by using 0.85% sterile normal saline, and the suspension was adjusted at 0.5% MacFarland standard.

The suspension was swabbed uniformly over entire surface of a sterile Muller Hinton Agar (MHA) plate. The antibiotics discs were placed on inoculated plate no closer than 15 mm from the edge and 24 mm from center of discs. The plates were then left at room temperature for 15 minutes for pre-diffusion and then incubated at 37°C. Diameter of the zone of inhibition around the disc was measured to the nearest millimeter using a metal caliper and the isolate was classified as sensitive, intermediate and resistant according to CLSI 2019 [12]. Isolates were considered as multi drug resistant (MDR) if bacterial isolates are non-susceptible to greater than or equal to three or more antimicrobial categories [14]

Standard quality control ATCC strains with known minimum inhibitory concentration including *Staphylococcus aureus* ATCC 25923, Escherichia *coli* ATCC 25922 and *Pseudomonas aeruginosa* ATCC 27853 were included in each run.

### Data analysis

As a first step of data management, the collected data was checked for its completeness, unrecorded values and unlikely responses. Accordingly, data cleaning was done manually whenever such indications are encountered. The laboratory results were registered onto a well-designed laboratory registration log book.

#### Statistical analysis

Data from the questionnaire and laboratory registration book were entered to Epi info version 6 and then exported to SPSS version 25 for further analysis. Using the parameters in SPPS, descriptive statistics like frequency distribution and proportion were computed. The analyzed data were presented using appropriate table format based on the types of the variables. Logistic regression analysis was performed. The strength of association between dependent and independent variables were measured using Odd ratio [OR] and 95% CI. The current research established P-value < 0.05 as an indicator of statistical significance.

### Ethical approval

The ethical clearance and approval was secured from College of Health Sciences research and ethics review committee. Following the clearance and approval, a supporting letter from the research directorate office was written to Tikur Anbessa specialized hospital to obtain permission letter. A written informed consent was obtained from all study participants involved in the study. The result of the research kept confidential. Confidentiality was maintained by numeric coding of samples and questionnaires. The objectives of the study and its benefits explained to the participants. Those identified positive for bacteriuria during the study period results were communicated to attending physicians and treated by appropriate drugs in line with the national guidelines for treatment.

## Results

### Sociodemographic characteristics

In this study, a total of 292 female cancer patients were enrolled. The mean age of study participants was 46.55±13.21 years, range 18–85 years. Most of the study participants were found in the age group of 38–45 which accounts 65 (22.3%), followed by age group 46–53 accounts 63 (21.6%). The majority of study subjects had no formal education 179 (61.3%), and 188 (64.4%) had housewife as their occupation (Table 1).

**Table 1:** Socio-demographic characteristics of study participants at TASH, Addis Ababa, Ethiopia.

Characteristics		Frequency	Percentage
Age group	18–21	7	2.4
	22–29	20	6.8
	30–37	51	17.5
	38–45	65	22.3
	46–53	63	21.6
	54–61	47	16.1
	62–69	25	8.6
	70–77	11	3.8
	78–85	3	1.0
Residence	Urban	152	52.1
	Rural	140	47.9
Religion	Orthodox	201	68.8
	Muslim	57	19.5
	Protestant	31	10.6
	Other	3	1.0
Education status	Illiterate	179	61.3
	Read and Write	12	4.1
	Elementary (1–8)	43	14.7
	High School	37	12.7
	Certificate	7	2.4
	Degree and Above	14	4.8
Occupation	House Wife	188	64.4
	Government	25	8.6
	Private Employee	17	5.8
	Merchant	4	1.4
	Farmer	36	12.3
	Student	7	2.4
	House Maid	2	.7
	Commercial Sex worker	1	.3
	No job	10	3.4
	Other	2	.7
Monthly income	< = 500	240	82.2
	501–1000	22	7.5
	1001–1500	6	2.1
	>1500	24	8.2
	Total	292	100.0

### Clinical characteristics of study participants

Out of 292 cancer patients, 40 (13.7%) had hematological malignancy and 252 (86.3%) wee solid organ malignancy patients respectively. Among solid malignancy, cervical cancer was the predominant proportion which accounts 107 (36.6%) followed by breast cancer 58 (19.9%). Majority of the solid cancer patients had clinical stage I which was 136 (46.6%), followed by clinical stage II which was 87(29.8) and most of them 244 (83.6%) has localized progression of cancers. Very few study participants had a history of smoking, family history of cancer and experience of cancer screening as shown in Table 2.

**Table 2:** Clinical characteristics of cancer patients at TASH, Addis Ababa, Ethiopia.

Clinical profiles		Frequency	Percentage
**Type of Cancer**	Hematological	40	13.7
	Solid	252	86.3
Solid	Breast	58	19.9
	Pharyngeal	12	4.1
	Cervical	107	36.6
	Colorectal	17	5.8
	Other	98	33.6
Stages of solid cancer	Grade I	136	46.6
	Grade II	87	29.8
	Grade III	45	15.4
	Grade IV	24	8.2
Progression of cancer	Localized	244	83.6
	Disseminated	48	16.4
History of hospitalization	Yes	118	40.4
	No	174	59.6
Duration of hospitalization	< = 3mnt	90	76.3
	>3mnt	5	4.2
History of taking antibiotics in six last month	Yes	88	30.1
	No	204	69.9
History of catheterization	Yes	3	1.0
	No	289	99.0
Family history of cancer	Yes	3	1.0
	No	289	99.0
Experience of cancer screening	Yes	3	1.0
	No	289	99.0
Experience of smoking	Yes	3	1.0
	No	289	99.0
Living with other smokers	Yes	19	6.5
	No	273	93.5
History of surgical incision	Yes	92	31.5
	No	200	68.5
Total		292	100.0

One hundred eighteen (40.4%) of the study participants had a history of hospitalization. Of which 90 (72.3%) had less than three months of hospitalization. Ninety-two (31.5%) of the participants had history of surgical incision. None of the study participants was working experience in chemical industry or radiation. Eighty-eight (30.1%) of the study participants had a history of taking antibiotics in the last six months. None of the clinical features of the study participants were associated with the urinary tract infections.

### Bacterial profiles and site of isolation

Of 292 cancer patients included in this study, 18 (.6.2%) were positive for bacterial culture. The isolates were *E*. *coli* 44.4% (8), *K*. *pneumoniae* 22.2% (4), *Citrobacter diversus* 16.7% (3), *Providentia rettgeri* 11.1% (2), and *Enterobacter cloacae* 5.6% (1). Polymicrobial growth was not detected in any of the clinical specimen (Table 3).

**Table 3:** Bacterial profiles among cancer patients at TASH, Addis Ababa, Ethiopia.

Bacterial profiles		Frequency	Percentage
Bacterial growth	Positive	18	6.2
	Negative	274	93.8
Bacterial isolates	*E*.*coli*	8	44.4
	*Klebsiella pneumonia*	4	22.2
	*Citrobacter diversus*	3	16.7
	*Providentia rettgeri*	2	11.1
	*Enterobacter cloacae*	1	5.6

### Antimicrobial resistance pattern and multidrug resistance (MDR)

In this study, high resistance rates were observed to Ampicillin 12 (66.7%), Augmentin 11(61.1%), and Ceftriaxone 10 (55.6%). On the other hand, a low resistance rate was detected for Meropenem 2(11.1%), Ciprofloxacin 3 (16.7%), Nitrofurantoin 2 (11.1%) and Piperacillin-Tazobactam 3(16.7%). *E*. *coli* isolates were resistant at least for one antibiotics tested. But, the antibiotic susceptibility results showed meropenem and nitrofurantoin as the most effective antibiotics for *E*.*coli* isolates. Gentamycin, meropenem, ciprofloxacin, and nitrofurantoin were the most effective antibiotics for *K*. *pneumoniae* isolates. However, 75% of *K*. *pneumoniae* isolates were resistant for Trimethoprim-Sulfamethoxazole and ampicillin. Fifty percent of the isolates of *Providentia rettgeri* were resistant for meropenem, chloramphenicol and Nitrofurantoin but completely resistant for Augmentin, Ampicillin, and Ceftriaxone. *Citrobacter diversus* isolates were completely resistant for Augmentin and 66.7% resistant for Ampicillin, Cefepime, and Ceftriaxone as shown in Table 4.

**Table 4:** Antimicrobial resistance pattern of isolates from cancer patients at TASH, Addis Ababa, Ethiopia.

Antimicrobial tested		Isolates, n = 18 (%)					Total
Classes	Antibiotics	*E*. *coli*, n = 8	*K*.*pneumoniae*, n = 4	*C*.*diversus*, n = 3	*P*.*rettgeri*, n = 2	*E*.*cloacae*, n = 1	n (%)
Aminoglycosides	GM	4 (50)	0	1 (33.3)	0	0	5(27.8)
Sulfonamides	SXT	5(62.5)	3(75)	1(33.3)	0	0	9(50)
Chloramphenicol	CHL	3(37.5)	2(50)	1(33.3)	1(50)	0	7(38.9)
Quinolones	CIP	2(25)	0	1(33.3)	0	0	3(16.7)
Nitrofurans	FM	1(12.5)	0	0	1(50)	0	2(11.1)
Beta lactam	Am	4(50)	3(75)	2(66.7)	2(100)	1(100)	12(66.7)
	CAX	5(62.5)	1(25)	2(66.7)	2(100)	0	10(55.6)
	FEP	5(62.5)	1(25)	2(66.7)	1(50)	0	9(50)
	CAZ	2(25)	2(50)	0	0	0	4(22.2)
	MEN	1(12.5)	0	0	1(50)	0	2(11.1)
	TZP	2(25)	1(25)	0	0	0	3(16.7)
	AUG	3(37.5)	2(50)	3(100)	2(100)	1(100)	11(61.1)

Abbreviations: GM- Gentamycin, SXT-Trimethoprim-Sulfamethoxazole, CIP- Ciprofloxacin, TZP-Piperacillin-Tazobactam, CHL-Chloramphenicol, FM- Nitrofurantoin, CAX-Ceftriaxone, CAZ Ceftazidime, AM-Ampicillin, MEN- Meropenem, FEP- Cefepime, AUG- Augmentin.

Multidrug resistances were detected in 33.3% of bacterial isolates, while one (12.5%) bacterial isolates were sensitive to all antibiotics tested. None of the isolated bacterial pathogens were resistant for all antibiotics classes tested. However, the result of MDR patterns compared within the species showed that 50% of *E*. *coli* isolates were MDR and 33.3% of *Citrobacter diversus* isolates were MDR, followed by *Klebsiella pneumoniae* (25%). All recovered isolates of *Providentia rettgeri* were resistant at least for two class of antibiotics as shown in Table 5.

**Table 5:** Multidrug resistance patters of bacterial isolates from cancer patients at Tikur Anbessa Specialized Hospital, Addis Ababa, Ethiopia.

Bacterial isolates	Antibiogram pattern, n (%)						
	R_0_	R_1_	R_2_	R_3_	R_4_	R_5_	MDR
*E*.*coli*	1(12.5)	0	3(37.5)	1(12.5)	2(25)	1(12.5)	4(50)
*Klebsiella pneumoniae*	0	0	3(75)	1(25)	0	0	1(25)
*Citrobacter diversus*	0	2(66.6)	0	0	0	1(33.3)	1(33.3)
*Providentia rettgeri*	0	0	2(100)	0	0	0	0
*Enterobacter cloacae*	0	1(100)	0	0	0	0	0
**Total**	1(5.6)	3(16.7)	8(44.4)	2(11.1)	2(11.1)	2(11.1)	6(33.3)

Note: R_0_: sensitive for all class of antibiotics, R_1_: resistant for one class of antibiotics, R_2_: resistant for two class of antibiotics, R_3_: resistant for three class of antibiotics, R_4_: resistant for four class of antibiotics, R_5_: resistant for five class of antibiotics, MDR-multidrug resistance.

## Discussion

The management of urinary tract infections in patients with cancer is a priority of public health due to its rapid onset and high level of morbidity and mortality [14]. Due to diverse nature of urinary tract infections etiology and antibiotic resistance patterns in periodic intervals, routine surveillance is needed to prevent the occurrence and transmission of urinary tract infections pathogens [15–17]. In the present study, we analyzed the distribution and antibiotic resistance of bacterial pathogens isolated from urinary tract infection from cancer patients at Tikur Anbessa specialized hospital cancer center.

In this study, the culture positivity rate was 18/292 (6.2%) among cancer patients. This finding was significantly lower with comparison with those that were previously reported in Gondar, Addis Ababa, and Egypt [14, 18, 19]. This variation could be study area difference since cancer patients relatively have close supervision and they get better treatment regimens.

*E*. *coli* (44.4%) was the most common organism isolated in cancer patients with UTI. This finding was in agreement with the study conducted in Egypt (30%) and India (40%) [19, 20]. However, this finding was lower (60.6%) than the study documented in Iranian cancer patients [21] and higher than (21.2%) the study recorded in Gondar [14]. The second frequently isolated gram-negative bacteria were *Klebsiella pneumonia* (22.2%) which is in lined with the study documented by Ashour and El-Sharif [19]. The observed discrepancy could be explained as due to geographic variation, sample size, study population and source of infections.

Intriguingly, we did not identify any gram positive bacteria and *Pseudomonas aeruginosa* from gram negative bacteria in our study participants even if these uropathogen were a commonly isolated as etiologic agent of UTI [21–23].

Many demographic factors and clinical features were analyzed as risk factors for bacteriuria in cancer patients in this study, however, no evidence was found to support the association between bacteriuria and demographic and clinical features of the cancer patients (P > 0.05). Correspondingly, in other studies [24–26] reported that neither of the factors and clinical features mentioned in our study had no effect on the occurrence of bacteriuria.

In our study, the estimated rate of MDR isolates was 33.3%, while 12.5% of the isolates were sensitive to all antibiotics tested which is in lower than the study documented 46.5% respectively) in Gondar cancer patients (14) and (54.4%) in Egyptian cancer patients [19].

In our study, 50% of *E*. *coli* isolates were Multidrug resistance. This finding was in line with the study conducted in Gondar, Egypt and India [14, 19, 20].

In this study, the highest resistance was recorded in Augmentin (61.1%), Ampicillin (66.7%) and Ceftriaxone (55.6%). This finding is in line with the study documented in Addis Ababa, Augmentin (80%), and Ceftriaxone (73.3%) respectively [18].

Our results showed that Meropenem and nitrofurantoin as the most effective antibiotics toward *E*. *coli* isolates. This finding was in agreement with the study documented in Addis Ababa cancer patients. However, higher resistance proportion for Trimethoprim-Sulfamethoxazole was documented [18].

Whereas, Gentamycin, Meropenem, Ciprofloxacin, and Nitrofurantoin were the most effective antibiotics for *K*.*pneumoniae* isolates. This finding is in agreement with the study previously reported in Iranian cancer patients [18].

As a limitation of this study, though in immunocompromised patient like cancer patients, the fungal agents especially Candidia are the most common etiologic agent of UTI, we did not test for the fungal agents as the causative agent of UTI due to the constraint of laboratory consumables. In addition, we did not test extended beta lactamase producing bacterial isolates which are the main sources of drug resistances.

### Conclusion

The overall burden of bacterial infections among cancer patients is considerably high. The most common bacterial isolates were *E*. *coli* and *K*. *pneumoniae*. This study finding showed that a significant distribution of MDR gram negative bacteria which may increase the burden of urinary tract associated infections in cancer patients.

## Supporting information

S1 File Questionnaire used to gather risk factors associated with urinary tract infection in cancer patients. (DOCX)

## Abbreviations

ATCC: American Type Culture Collection
CLSI: Clinical and Laboratory, Standard Institute
MDR: Multi Drug Desistance
SPSS: Statistical Package for Social Science
UTI: Urinary Tract Infection
