# Supplementary material for: Urinary tract infection in cancer patients and antimicrobial susceptibility of isolates in Tikur Anbessa Specialized Hospital, Addis Ababa, Ethiopia
Source: PLoS One. 2020 Dec 10;15(12):e0243474. doi: 10.1371/journal.pone.0243474 (PMC7728278; doi:10.1371/journal.pone.0243474)
Supplement: S1 File — (DOCX) [file pone.0243474.s001.docx]

## Additional information 1. Questionnaire used to gather risk factors associated with Urinary tract infection in cancer patients

Date: _______________________

**Title of the study:** “**Urinary tract infection in cancer patients and antimicrobial susceptibility of Isolates in Tikur Anbessa specialized Hospital, Addis Ababa, Ethiopia”**

**Direction for the data collector: Please encircle the participants’ response accordingly and put words or phrases on the space given.**

**Pregnant registration/card number__________________**

**Date of interview______________________**

**Data collector/Interviewer Name__________________________**

**Name of Health Centre___________________________________**

**Part 1: Questions used to asses Soci-demographic characteristics and clinical features**

|  | **Questions** | **Response** |
| --- | --- | --- |
| 101 | Age in years | **______________** |
| 102 | Sex of participants | 1. Male 2. Female |
| 101 | Residence | 1. Urban 2. Rural |
| 103 | Religion | 1. Orthodox 2. Muslim 3. Protestant 4. Other (specify) |
| 104 | Educational status | 1. Illiterate 2. Read and write 3. Elementary (1-8) 4. High school (9-12) 5. Certificate 6. University degree and above |
| 105 | What is your occupational status | 1. House wife 2. Government employee 3. Private employee 4. Merchant 5. Farmer 6. Student 7. House maid 8. Commercial sex worker 9. No job 10. Others (specify)_________ |
| 106 | What is your monthly income (in Eth birr) | -------------------------------- |
| 107 | Family history of cancer | 1. Yes 2. No |
| 108 | Current smoking history | 1. Yes 2. No |
| 109 | Type of underlying cancer | 1. Hematologic Malignancy 2. Solid tumor |
| 110 | Treatment administration for cancer | 1. Yes 2. No |
| 111 | If yes for Q 110, which type of treatment you have been taking? | 1. Adjuvant or neoadjuvant treatment 2. First-line treatment 3. Second (subsequent)-line treatment 4. Bone marrow transplant (BMT) 5. Others----------------------------------------- |
| 111 | Have you been admitted before in hospital in the last one year? | 1. Yes 2. No |
| 112 | If yes for Q 111, for how long did you stay in hospital? | 1. > 3 months 2. < 3 months |
| 113 | Did you take antibiotics in the last 6 months? | 1. Yes 2. No |
| 114 | Surgical incision | 1. Yes 2. No |
| 115 | Presence of a clinical site of infection | 1. Yes 2. No |
| 116 | If yes for Q 115,what type of infection has been observed? | 1. Mucosa 2. Lower respiratory tract 3. Skin/soft tissue 4. Vascular access device 5. Urinary tract/drainage device 6. Upper respiratory tract 7. Gastrointestinal tract 8. Others |
| 117 | History of catheterization or other medical device implantation | 1. Yes 2. No |
| 118 | Stage of cancer | 1. Grade I 2. Grade II 3. Grade III 4. Grade IV |
| 119 | Cancer progression | 1. Localized 2. Disseminated |
